# Current Understanding of the Structure, Stability and Dynamic Properties of Amyloid Fibrils

**DOI:** 10.3390/ijms22094349

**Published:** 2021-04-21

**Authors:** Eri Chatani, Keisuke Yuzu, Yumiko Ohhashi, Yuji Goto

**Affiliations:** 1 Graduate School of Science, Kobe University, 1-1 Rokkodai, Nada, Kobe, Hyogo 657-8501, Japan; 192S227S@stu.kobe-u.ac.jp (K.Y.); yumiko_oha@dolphin.kobe-u.ac.jp (Y.O.); 2 Global Center for Medical Engineering and Informatics, Osaka University, 2-1 Yamadaoka, Suita, Osaka 565-0871, Japan; gtyj8126@protein.osaka-u.ac.jp

**Keywords:** protein, amyloid, structure, stability, polymorphism, spaciotemporal control, reversibility

## Abstract

Amyloid fibrils are supramolecular protein assemblies represented by a cross-β structure and fibrous morphology, whose structural architecture has been previously investigated. While amyloid fibrils are basically a main-chain-dominated structure consisting of a backbone of hydrogen bonds, side-chain interactions also play an important role in determining their detailed structures and physicochemical properties. In amyloid fibrils comprising short peptide segments, a steric zipper where a pair of β-sheets with side chains interdigitate tightly is found as a fundamental motif. In amyloid fibrils comprising longer polypeptides, each polypeptide chain folds into a planar structure composed of several β-strands linked by turns or loops, and the steric zippers are formed locally to stabilize the structure. Multiple segments capable of forming steric zippers are contained within a single protein molecule in many cases, and polymorphism appears as a result of the diverse regions and counterparts of the steric zippers. Furthermore, the β-solenoid structure, where the polypeptide chain folds in a solenoid shape with side chains packed inside, is recognized as another important amyloid motif. While side-chain interactions are primarily achieved by non-polar residues in disease-related amyloid fibrils, the participation of hydrophilic and charged residues is prominent in functional amyloids, which often leads to spatiotemporally controlled fibrillation, high reversibility, and the formation of labile amyloids with kinked backbone topology. Achieving precise control of the side-chain interactions within amyloid structures will open up a new horizon for designing useful amyloid-based nanomaterials.

## 1. Introduction

Self-assembly is a fascinating process that enables materials to spontaneously form organized nanostructures. Biological organisms are one of its ultimate outcomes, and inside them various molecules form complexes with sophisticated functions. Among the biomacromolecules constituting living systems, proteins have outstanding ability to assemble themselves into various structures, ranging from multimers with relatively small number of subunits to larger complexes with characteristic shapes such as cages, rods, sheets, or more complicated ones. There are also supramolecular protein polymers in which periodic assemblies are formed almost infinitely, like actin filaments and crystals. A wide range of protein complex structures have been characterized so far, and the accumulated data have inspired researchers to understand principles of protein self-assembly [1] and to design new proteins or peptides to pursue desired nanostructures and functions [2,3,4].

Among the various types of proteins self-assemblies, amyloid fibrils are one of the structures that are naturally formed in living organisms. They are classified as supramolecular polymers, with β-strands stacked perpendicularly to the long axis of the fibril [5]. Amyloid fibrils were originally found in pathological deposits, and have been thought to be an aberrant protein state associated with human diseases. However, it is currently considered that amyloid structures are not always formed as a product of protein misfolding, as it was later revealed that microorganisms and even higher eukaryotes sometimes produce amyloid-like structures for biological purposes [6,7]. It is now recognized that the property of forming amyloid fibrils is observed not only in the amyloidogenic protein associated with diseases, but also in a wide variety of proteins and peptides, indicating that amyloid formation is a common property shown by polypeptide chains [8].

In contrast to many protein assemblies that can form intermolecular interactions only when each polypeptide chain folds into a higher-order structure, the amyloid structure is based on interactions among polypeptide main chains. Therefore, the folding of each constituent protein or peptide into a particular tertiary structure is not a prerequisite for the formation of amyloid fibrils. This simple architecture facilitates the use of amyloid folds as one of the building blocks for the de novo design of polypeptide-based nanomaterials. In addition, the nanomechanical properties of amyloid structures is also important for designing nanomaterials. Experimental and in silico studies have suggested that individual fibrils, largely from the dense backbone hydrogen-bonding network, commonly possess high stiffness with an elastic modulus in the GPa range [9]. Among many advances in the design of many β-based supramolecular structures [10], amyloid-based nanofibers have also been reported [11,12,13]. There is also a successful example of the formation of sheets in which individual fibrils are stacked perpendicular to the fibril axis [14]. Based on such recent trends in protein and peptide engineering, a comprehensive understanding of detailed properties of amyloid assembly is expected to contribute not only to the prevention and treatment of amyloid-related diseases but also to exploring the functionality and furthermore, the development of new nanomaterials for biological functions.

In this review article, we address recent advances in understanding the architecture of amyloid fibrils to explore the potentials of engineering applications. Many studies have been conducted on the structure, stability, and formation mechanisms of amyloid fibrils, and they have provided a great amount of valuable information to date. With reference to previous reports, we summarize the current understanding of the structural architecture of amyloid fibrils, which will help provide the inspiration for new protein and peptide designs.

## 2. Fundamental Structure of Amyloid Fibrils

**Figure 1:**
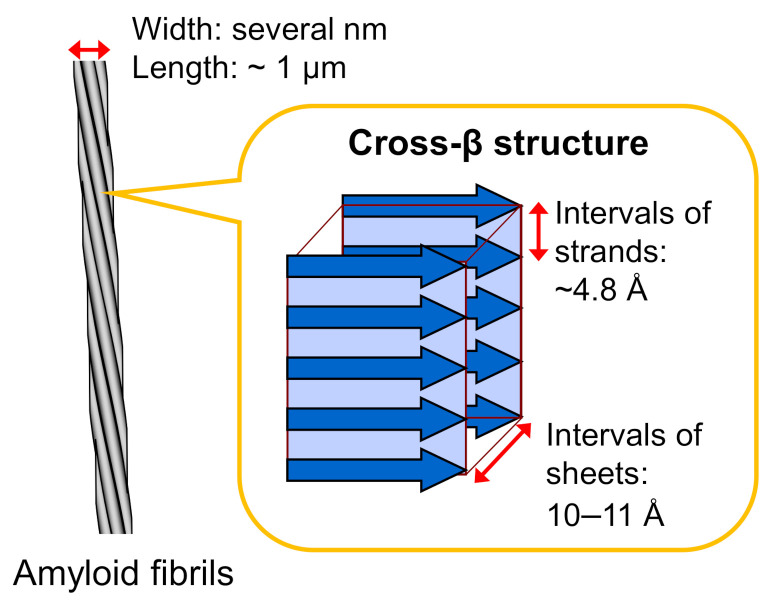
Schematic illustration of the structure of an amyloid fibril. Amyloid fibrils typically show needle-like and unbranched morphology, which consists of several protofilaments a few nanometers in width and around a micrometer in length that are laterally bundled. Each protofilament shows a cross-β structure, where β-strands are stacked perpendicular to the long axis of the fibril.

Amyloid fibrils typically have a hierarchical structure in which several protofilaments a few nanometers in width and around a micrometer in length are laterally bundled. Each protofilament consists of a cross-β structure with β-strands stacked perpendicular to the long axis of the fibril through a hydrogen-bonding network (Figure 1). This basic structure was initially revealed in the 1950s by studies of X-ray fiber diffraction and electron microscopy [15,16], and the elucidation of more detailed structural properties has progressed since then by applying various physicochemical methods. In the early stages of structural research, the cross-β structured regions within the constituent polypeptide chains were identified by using limited proteolysis or hydrogen/deuterium exchange in combination with NMR or mass spectrometry [17,18].

**Figure 2:**
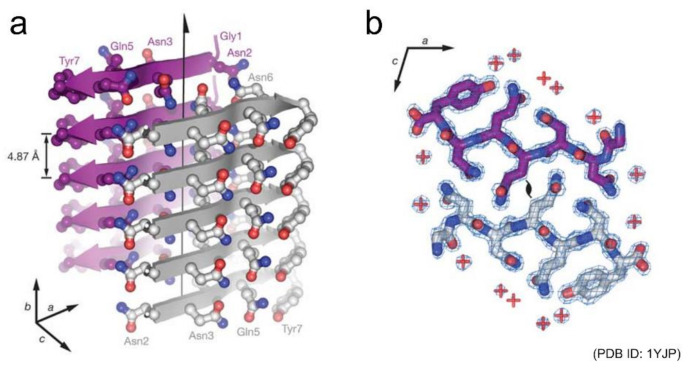
Structure of a steric zipper formed by the short peptide GNNQQNY. (**a**) Side view and (**b**) top view of the pair of β-sheets are shown. In (**a**), the backbone of each β-strand is represented as an arrow, and the side chains protruding from the backbones are shown as ball-and-stick models. In (**b**), two GNNQQNY molecules are shown as stick models, and their interface demonstrates the shape complementarity of the asparagine and glutamine side chains. Reprinted from [19] with permission. Copyright (2005) Springer Nature.

Shortly after those early studies, Eisenberg and co-workers succeeded in determining the atomic structures of a seven-residue peptide segment GNNQQNY from yeast prion Sup35 and other short peptides by X-ray crystallography using microcrystals [19,20]. The structures of multiple kinds of peptides revealed that peptides generally stack extended β-strands in a register to form a β-sheet, and that a pair of β-sheets forms a steric zipper with side chains interdigitated tightly (Figure 2). Peptide dimers packed in a similar manner to steric zippers were also found from the cryo-electron microscopy (cryo-EM) of amyloid fibrils of a twelve-residue fragment from an amyloidogenic immunoglobulin (IgG) light chain [21]. Both the polar and non-polar side chains participate to form the steric zipper, and van der Waals interactions seem to contribute dominantly to its stability. The tight packing is common to steric zippers formed by various peptides, which strongly indicates that side-chain interdigitations are important for the stability of amyloid fibrils and the formation of backbone hydrogen bonds.

**Figure 3:**
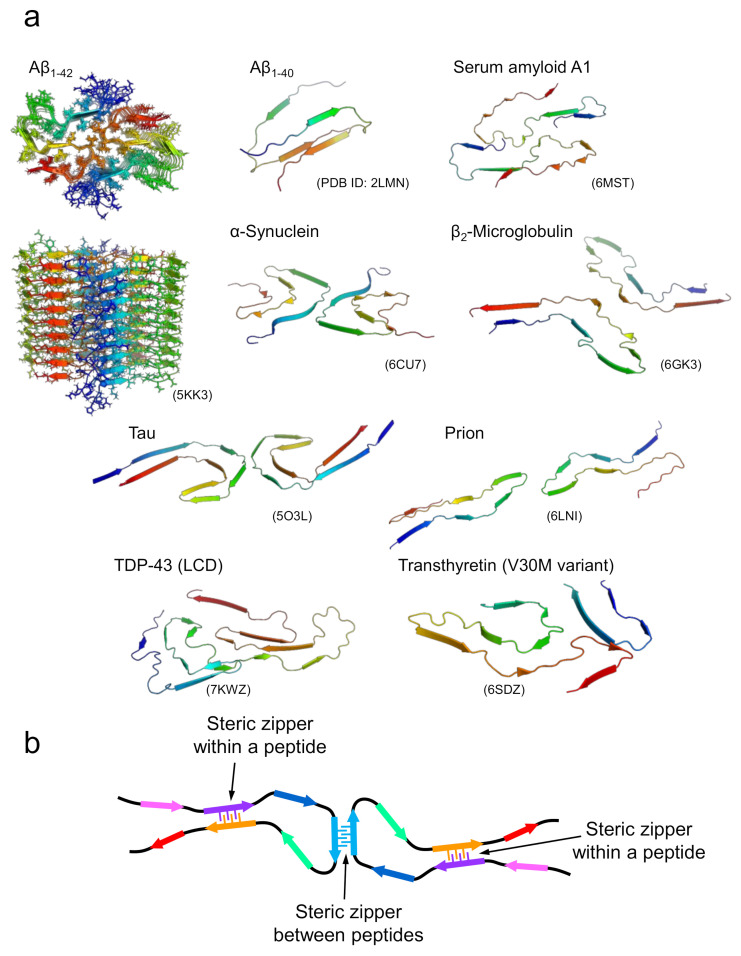
Structures of amyloid fibrils composed of longer polypeptide chains. (**a**) A gallery of structures of amyloid fibrils formed by proteins. For Aβ_1-42_, the top and side views of multiple polypeptide chains that make up an amyloid fibril are displayed. For the remaining proteins, the three-dimensional structures of polypeptide chains that make up a single layer along the fibril axis are displayed. (**b**) A schematic image representing the fundamental architecture of amyloid fibrils composed of long polypeptide chains.

In the case of amyloid fibrils formed with longer polypeptide chains, it is unlikely that all side chains are packed tightly and complementarily as observed in the steric zippers of short peptides. Although their detailed structural properties were unknown until recently, advances in cryo-EM and solid-state NMR techniques have increased the number of studies showing the atomic structures of the amyloid fibrils formed by disease-related proteins dramatically over the past few years [15,22,23,24,25,26,27,28]. The determined structures have clearly shown that several pairs of β-sheets are formed both within a peptide and between different peptides (Figure 3a). Interestingly, polypeptide chains are “folded” into relatively complex structures in all cases, although the conformation per polypeptide is fundamentally planar, which is contrastive to globular native folds. In the majority of amyloid structures, it has been determined that the polypeptide chains form several β-strands linked by loops. In some parts, the β-strands are stabilized with side-chain interactions in a manner similar to that of steric zippers between various pairs of β-strands within the polypeptide chain and, when the fibril is composed of multiple protofilaments, between different polypeptide chains (Figure 3b) [15]. While steric zippers are usually formed between two identical polypeptides in the case of short peptides, they are formed between two different sheets with non-identical sequences in the majority of cases of long polypeptides, which are referred to as hetero-steric zippers [29]. These structural features suggest that the tight packing of side chains seen in the steric zippers in short peptide segments contributes substantially to the structural stabilization of amyloid fibrils in the case of long polypeptides. Consistent with this idea, direct measurement of thermodynamic parameters of amyloid formation by isothermal titration calorimetry using β_2_-microglobulin revealed a negative heat capacity change similar to that found in native folding, suggesting that hydrophobic interactions are significantly involved in amyloid structure [30].

On the other hand, the formation of steric zippers is localized, and the side chains in the other regions appear to be relatively loosely packed. This feature was also implicated from the significantly large value of the partial specific volume obtained using a densitometer [31], and from the effect of hydrostatic pressure on β_2_-microglobulin, in which the original fibril structure changed to a more packed one with smaller volume under high pressure [32]. A previous amylome study estimating the number of different segments with high propensity to form steric zippers in the open reading frames of genomes also suggested that sequences with high propensity to form steric zippers do not cover the whole region of the polypeptide sequence, but merely the one or more short segments that are localized within it (Figure 3b) [33]. Such structural features are compatible with the view that amyloid fibrils are a main chain-dominated structure with hydrogen bond networks of the peptide backbone, in contrast to the globular native structure of proteins, represented as a side chain-dominated structure evolved by pursuing a unique fold, with the optimal packing of side chains [34,35]. The weaker contribution of side chains other than steric zippers rationally explains the planar structure of the polypeptide chain within the fibril, and the similar morphology of amyloid fibrils among various proteins with different amino-acid sequences. Furthermore, this structural property is reminiscent of the manifestation of the plastic features of amyloid structure as revealed by the pressure-induced structural change, and, furthermore, polymorphism, as discussed below.

## 3. Polymorphism of Amyloid Fibrils

There have been accumulating studies showing that diverse amyloid structures are formed even from the same protein; this phenomenon is referred to as amyloid polymorphism [22,36]. Amyloid polymorphs are often produced in vitro, with properties depending on fibril growth conditions such as solvent [37], temperature [38], protein concentration [39], and agitation [40]. Recently, amyloid polymorphism has been studied as a molecular basis of pathologies, since it was reported that polymorphs of amyloid-β (Aβ) fibrils are observed in brain tissues from patients with different clinical subtypes of Alzheimer’s disease, as was demonstrated by analyses using fluorescent probes [41] and solid-state NMR [42]. Furthermore, polymorphs of tau filaments have been observed in both Alzheimer’s and Pick’s diseases by cryo-EM [23,24]. From these findings, amyloid polymorphism has been suggested to have a close relationship to the expression of various disease subtypes. Considering that the structural diversity in amyloid fibrils underlies their differences in properties such as growth rates, structural stability [37,38], and toxicity to cultured cells [40,43], understanding the structural principles of amyloid polymorphism will lead to the design of amyloid structures with controlled physicochemical characteristics. Recently, from the simulation of deformations with a coarse-grained model, different elastic moduli were suggested to exist among polymorphs of Aβ fibrils [44,45]. This finding provides molecular details on the stability of amyloid fibrils and helps to discuss fibrillation rate and fragility, which are thought to be closely related to cytotoxicity.

**Figure 4:**
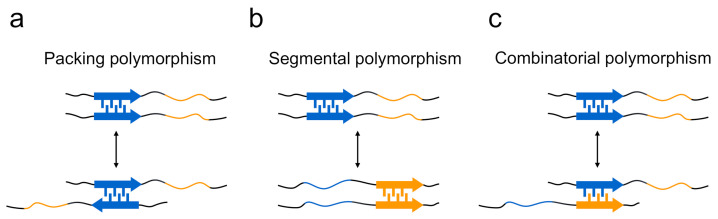
Three proposed models explaining the formation mechanism of polymorphs in terms of steric zippers. (**a**) Polymorphs are produced when steric zippers are formed with different packing patterns or with different interfaces between the same pair of segments (i.e., packing polymorphism). (**b**) In the case of polypeptide chains that contain several different segments with high propensity to form steric zippers, the difference in region of steric zippers is another mechanism for forming polymorphs (i.e., segmental polymorphism). (**c**) Additionally, differences in the sequences of paired polypeptide segments also contribute to the formation of polymorphs (i.e., combinatorial polymorphism).

The structural differences of amyloid polymorphs have been observed at various structural levels, including the core regions, secondary structure, protofilament arrangements, and morphology of the amyloid, and the structural basis of the occurrence of amyloid polymorphism has been elucidated. In terms of the steric zippers shown by short segments, Eisenberg and co-workers have proposed a rational hypothesis suggesting that the diversity of steric zippers is responsible for various amyloid structures [29,46]. In their studies of the microcrystals of short segments of islet amyloid polypeptide (IAPP), tau, Sup35, and Aβ, it was revealed that the same short segments occasionally form steric zippers with different packing patterns of two sheets, from which the packing polymorphism within steric zippers has been proposed as one of mechanisms to explain that a single protein produces the structural diversity of amyloid fibrils (Figure 4a).The typical form of the packing polymorphism is the difference in interdigitation of the two segments, which is actually observed in the segments of, for example, ^28^SSTNVG^33^ from IAPP and ^16^KLVFFA^21^ from the Aβ peptide, and there is also another type of packing polymorphism in which the orientation of their faces is different [47,48]. Packing polymorphism was also verified by a solid-state NMR analysis of amyloid fibrils formed by a short peptide segment from yeast prion Sup35, in which three distinct intermolecular packings between the constituent peptides were demonstrated [49]. Furthermore, differences in the regions of steric zippers, i.e., segmental polymorphism, have also been proposed from the observation that many amyloidogenic proteins, including Sup35 [20], IAPP [47], and Aβ [48], contain multiple different segments with the potential to form a steric zipper (Figure 4b). In agreement with this proposed model, the NM domain of Sup35 (Sup35NM) formed amyloid fibrils with a different core region by the mutation of Ser17 to arginine [50]. The differences in the combination of paired polypeptide chains, which is referred to as combinatorial polymorphism, have also been proposed, based on the idea that peptide sequences do not have to be the same to achieve tight packing of side chains (Figure 4c) [47], although it is difficult to observe them directly in peptide microcrystals.

**Figure 5:**
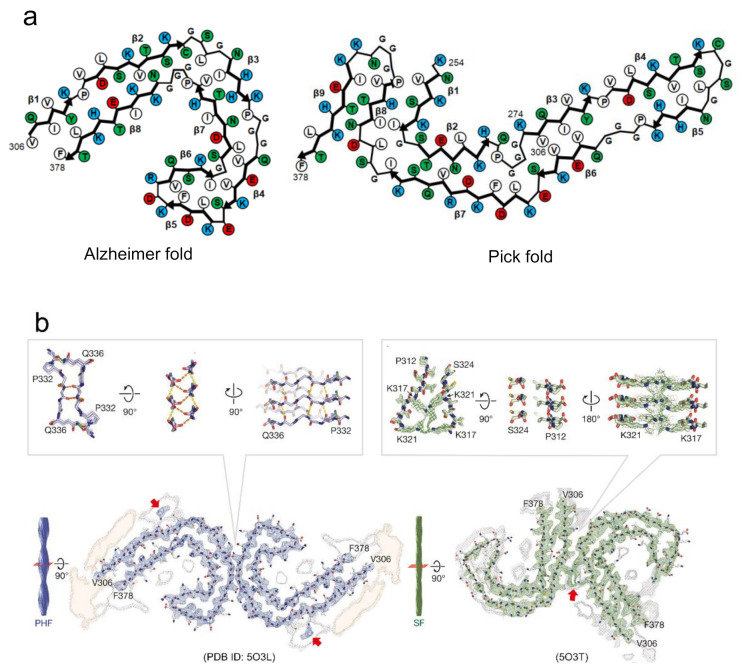
Proposed mechanism of the formation of polymorphs based on the diversity of steric zippers. (**a**) Tau amyloid polymorphs from Alzheimer’s and Pick’s disease patients, showing difference in protofilament folds. Schematic illustrations of the arrangements of side chains in this panel are redrawn based on those in [23,24]. Hydrophobic, polar, positively charged, and negatively charged residues are colored by white, green, blue, and red, respectively. (**b**) Tau amyloid polymorphs from Alzheimer’s patients, PHF and SF, showing differences in the arrangement of interactions of two protofilaments. Red arrows indicate electron densities attributed to the interaction with the side chains of K^317^ and K^321^. Reproduced with modification from [23], with permission. Copyright (2017) Springer Nature.

Recently, the detailed structures and interactions underlying amyloid polymorphism have revealed by atomic structures of various proteins [15,26]. Overall, the structures of amyloid polymorphs can be characterized by diverse folds within a protofilament or different interactions between protofilaments. In the polymorphs of tau filaments from Alzheimer’s and Pick’s disease patients revealed by cryo-EM, two different protofilament folds were observed [23,24] (Figure 5a). Although both folds share a similar β-strand composition, different β-strands are paired to form hetero-steric zippers. As a result, the main chain shows different folding patterns with distinct turn conformations. A similar difference in the protofilament fold was also observed among the polymorphs of IAPP [51], among those of TAR DNA-binding protein 43 (TDP-43) [52], and between the rod and twister polymorphs of α-synuclein fibrils [25]. In the latter case, the protofilaments of both polymorphs commonly form ordered bent β-arch folds, whereas the twister polymorph additionally forms a Greek-key-like fold using some terminal residues. These observations indicate that the different folds of protofilaments are a fundamental factor in the formation of amyloid polymorphs.

There is another case where amyloid polymorphs can be produced by different arrangements of protofilaments even if the polypeptide folds are identical. This type explains the formation of amyloid polymorphs in many cases, as observed in tau [23], Aβ [53], and β_2_-microglobulin amyloid polymorphs [15]. In most cases, two protofilaments are arranged in a rotational symmetry. Among them, the paired helical and straight filaments of tau amyloid polymorphs from Alzheimer’s patients are typical example that the arrangement of interactions of two protofilaments are different [23] (Figure 5b). In the paired helical filaments (PHF), the two protofilaments with identical folds contact with helical symmetry at the interface of ^332^PGGGQ^336^. Although the continuous glycine residues at this interface appear to have difficulty achieving the tight packing of side chains, it was found that the interface is stabilized by backbone hydrogen-bonding, both within and between the two protofilaments. Additionally, two hydrogen bonds between the side chain of Q^336^ and the backbone carboxyl of K^331^ on the opposite protofilament. In the straight filaments (SF), the two protofilaments are packed asymmetrically between ^321^KCGS^324^ in first protofilament, and ^313^VDLSK^317^ in the second. These residues themselves do not seem to form any strong interactions. Instead, the protofilament interface appears to be stabilized by the interactions of the side chains in the vicinity of the interface of both protofilaments (i.e., K^317^, T^319^, and K^321^). The observation of tau amyloid fibrils may suggest that the inter-protofilament interactions are weaker than the intra-protofilament interactions. Furthermore, in some cases, a different number of protofilaments forming amyloid fibrils results in another type of polymorph. This case was observed in the polymorphs of Aβ40 [22,40,54] and in the narrow and wide filaments of tau fibrils from Pick’s patients [24]. From these findings, it has been indicated that the diverse patterns of protofilaments packing and arrangement due to the difference in intra- and inter-protofilament interactions, are responsible for the amyloid polymorphism. As more complicated structures, some of the revealed polymorphs show both different folds within protofilaments and different arrangements among protofilaments [52], although the number of applicable cases is small.

## 4. Kinetic and Thermodynamic Controls in Functional Amyloid Structures

It has become increasingly clear that amyloid structures are not only found in aberrant and disease-related aggregates, but also in functional protein assemblies referred to as “functional amyloids” [7]. Functional amyloids are mainly produced by prokaryotes [6,55] and some of them are produced by higher eukaryotes [56]. It is currently understood that functional amyloids serve diverse purposes, and some of them act as biofilm matrix proteins playing a scaffolding role in microorganisms [6,55]. In filamentous fungi, amyloid fibrils organize an amphipathic monolayer by assembling laterally on the surface of a hydrophobic/hydrophilic interface, thus reversing wettability, as seen in water-repellent spore coats [57]. There are also functional amyloids playing other roles such as virulence, protection against degradation and adhesion [55,58], as well as in the more complicated regulatory functions in the transcription, heterokaryon incompatibility, and long-term memory, etc. of eukaryotes, as have also been reported [56]. For amyloid fibrils to exert biological functions, the spatiotemporal regulation of their formation seems to be achieved elaborately. Since the structures of natural functional amyloids are thought to have been optimized through the process of molecular evolution, elucidating their structural properties will provide valuable insights for the use of amyloid structure in protein engineering.

**Figure 6:**
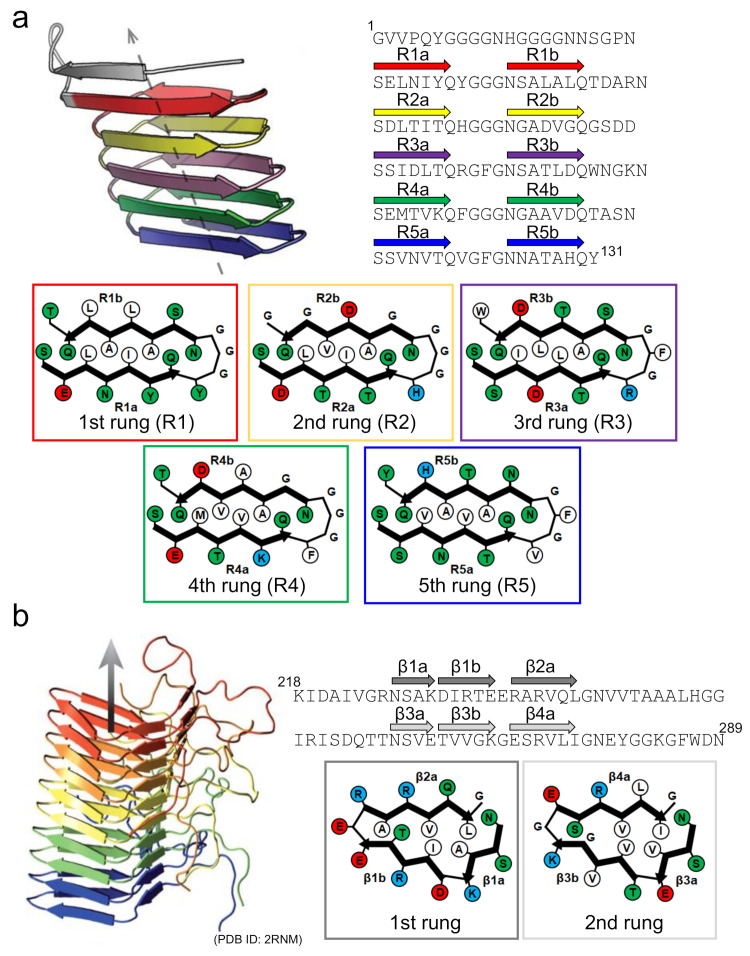
Structures of functional amyloids consisting of a β-solenoid structure. (**a**) The side view of the protofilament and the schematic arrangement of side chains within the protofilament of CsgA. The left-handed structure, which is one of the low-energy conformations predicted by the computer simulation, is shown as a representative example. The side view was reproduced from [64], with permission. Copyright (2014) American Chemical Society. The schematic arrangement of side chains was drawn with reference to [64]. A single polypeptide chain takes a 5-rung solenoid structure, and each rung is shown in a different color. In the schematic illustrations of the arrangements of side chains at each rung, hydrophobic, polar, positively charged, and negatively charged residues are colored in white, green, blue, and red, respectively. The R1b strand is possibly unstable compared to the other ones according to the simulated structure. (**b**) Side view of the protofilament and a schematic illustration of the side chain arrangements within the protofilament of HET-s(218-289). Reproduced with modification from [67], with permission. Copyright (2008) Springer Nature. In the side view, five polypeptide chains constituting a β-solenoid structure, each of which takes a 2-rung solenoid, are shown in different colors. In the schematic illustrations of the arrangements of the side chains at each rung, hydrophobic, polar, positively charged, and negatively charged residues are colored in white, green, blue, and red, respectively.

Curli is one of the functional amyloids serving as a scaffold in bacterial biofilms [6,55]. Since the discovery of the amyloid-like structure in curli produced by *Escherichia coli* by Chapman et al. [59], the biosynthesis mechanism of *E. coli* curli has attracted attention as an important example where the location and timing of formation are finely controlled [60]. The *E. coli* curli is composed mainly of CsgA, and its self-assembly to amyloid-like structure is mediated in vivo by another component, CsgB, localized on the cellular surface [61,62]. The amino-acid sequence of CsgB has a high degree of similarity with that of CsgA, and the cross-seeding of CsgA with CsgB is thought to be the molecular mechanism by which fibrillation is initiated in CsgA. Solid-state NMR and computational studies have suggested that both CsgA and CsgB form a β-solenoid (or β-helix) structure with five incomplete repeats, with a length of approximately 20 amino-acid residues [63,64]. With a Ser-X_5_-Gln-X_4_-Asn-X_5_-Gln consensus sequence contained within each repeat, CsgA and CsgB form five successive rungs made of two β-strands with a rectangular core, within which the number of contributing intramolecular contacts is higher than it is in the typical disease-related in-register β-sheet structure (Figure 6a).

Interestingly, several specific aspartic acid and glycine residues are observed in the internal repeats of CsgA that function as “gatekeepers” to suppress the spontaneous nucleation of CsgA itself [65]. It has also been proposed that CsgA shows negligible fibril self-replication through secondary nucleation processes [66]. Based on these findings, the β-solenoid fold could be regarded as one of the amyloid folds evolved for controlling the structure and formation processes. This view is supported by the structure of the prion-forming domain of the HET-s prion (HET-s(218-289)), a protein forming functional amyloids for the heterokaryon incompatibility system of *Podospora anserina*, whose C-terminal prion domain forms a β-solenoid structure with a triangular hydrophobic core [67] (Figure 6b). However, a similar β-solenoid structure has recently been reported as the infectious form of the mammalian prion responsible for prion diseases [68], in addition to the in-register β-sheet structure often seen in disease-related amyloid fibrils [69]. At present, the physiological priority of the β-solenoid structure remains somewhat puzzling [70], and future research must be conducted to reveal differences in the biological contributions of the β-solenoid and the in-register β-sheet structures.

There are also intriguing examples that some amyloidogenic proteins and peptides utilize an ability to switch between an amyloid and a soluble state. Hormone storage is a representative case, and the idea of using amyloid fibrils as depots of active proteins or peptides has been an attractive concept [71,72]. This idea has been raised since the first discovery by Maji et al. that peptide hormones are stored in secretory granules by taking amyloid-like structures [73]. While amyloid structures appear irreversible due to high stability, especially in diseases-related cases, they are depolymerizable, as they are constructed by non-covalent interactions. The depolymerization of amyloid fibrils has been observed upon incubation under protein denaturing conditions, such as at high temperature [74], and in the presence of chemical denaturants such as guanidium hydrochloride [75], urea [76], and dimethyl sulfoxide [77]. Understanding the structural properties by which amyloid fibrils can switch to a dissociated form will reveal an important property that endows functionality to amyloid structure.

**Figure 7:**
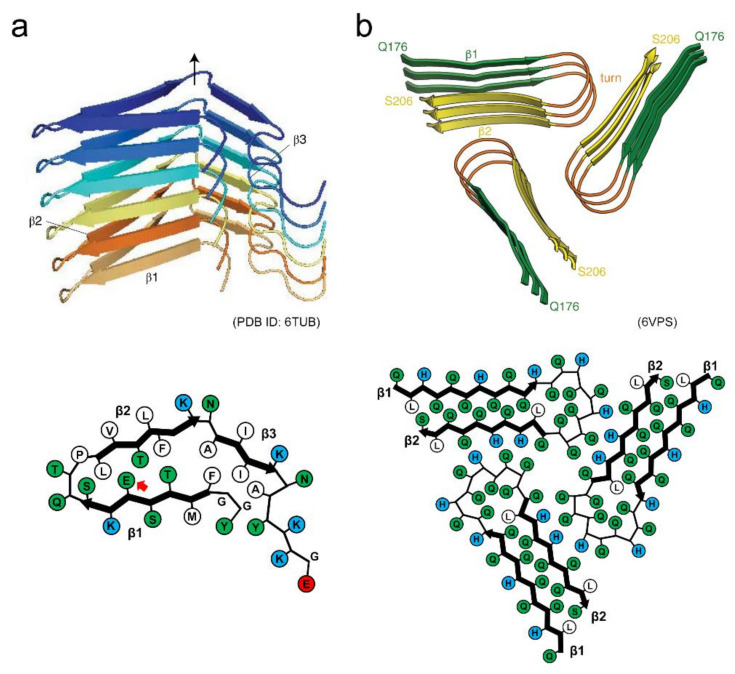
Structures of functional amyloids showing high reversibility. (**a**) Side view of the protofilament and schematic illustration of the arrangement of side chains within the protofilament of β-endorphin. Reproduced with modification from [27], with permission. Copyright (2020) Springer Nature Limited. Each polypeptide chain is shown in a different color. In the schematic arrangement of the side chains, hydrophobic, polar, and positively charged residues are colored in white, green, and blue, respectively. The red arrow indicates the glutamate residue with the higher p*K*a value. (**b**) Side view of the filament and schematic illustration of the arrangement of side chains within the filament of Orb2. While each polypeptide chain is not distinguished by color, each strand within the polypeptide chain is shown in a different color instead. In the schematic arrangement of side chains, hydrophobic, polar, and positively charged residues are colored in white, green, and blue, respectively. Reproduced with modification from [28], with permission. Copyright (2020) American Association for the Advancement of Science.

The structure of human hormone β-endorphin amyloid fibrils has recently been determined by solid-state NMR [27] (Figure 7a). Each β-endorphin molecule constructs a single layer of the solenoid made of three β-strands. The β1 and β2 strands interact through a hydrophilic core, and a glutamate residue is located within it. The β1 and β2 strands also form a hydrophobic core with the β3 strand. Together with other experimental analyses, it has been clarified that the glutamate residue in the hydrophilic core is still protonated in the secretory granule at pH 5.5 due to the environment-induced large shift of p*K*a. It is deprotonated upon the pH change to 7.4 (in blood), and then facilitates the destabilization and subsequent disassembly of the fibril core. The charge-induced destabilization is consistent with a previous report that amyloid fibrils of α-synuclein with negative charges are buried in the core showed low stability, sufficient to cause cold denaturation above 0 °C [78]. A molecular dynamic simulation study also showed that the mechanical and thermodynamic stability of α-synuclein amyloid fibrils are significantly lower than those of Aβ amyloid fibrils [45].

The amyloid structure of Orb2, a protein involved in long-lasting memories in the brain of *Drosophila* has also determined very recently by cryo-EM [28], providing valuable insights into structural basis of functional amyloids. In this structure, three identical protofilaments are packed in the threefold symmetry. Each protofilament is formed with glutamine-rich regions of the prion domain of Orb2, and adopts a hairpin-like structure with interdigitated glutamine residues (Figure 7b). In addition to the characteristic hydrophilic core composed of glutamine, three histidine residues that could have positive charges at physiological pH are found at the protofilament interface. Although there has yet been no direct evidence that Orb2 switches the assembly and disassembly of amyloid structures in vivo, the introduction of charged residues into the core or the protofilament interface may be one of the key ways to achieve the dynamic properties of amyloid structures.

## 5. New Attention to Loosely Packed Amyloid-Like Assemblies

**Figure 8:**
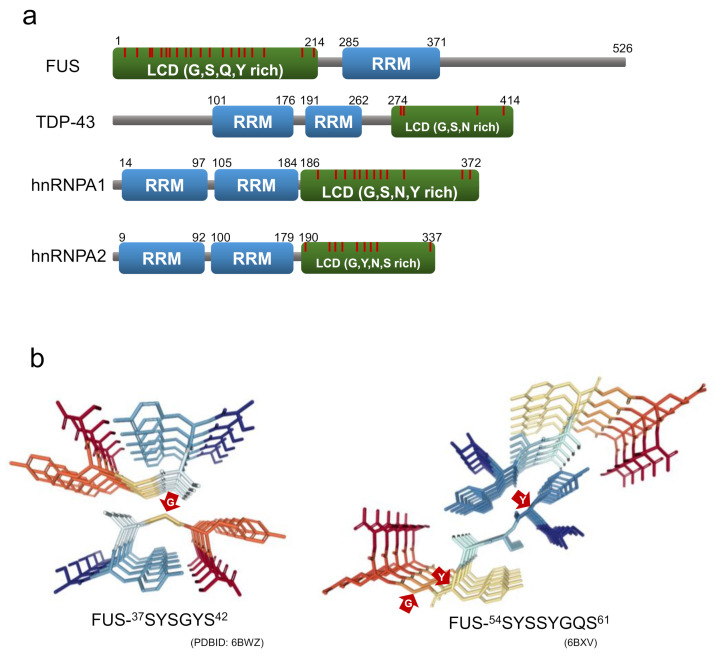
(**a**) Domain structure of RNA-binding proteins consisting of stress granules. LCD and RRM indicate a low-complexity domain and an RNA recognition motif, respectively. The red line is the position of the G/S-Y/F-G/S motifs. (**b**) Crystal structure of LARKS from FUS (residues 37-42 and 54-61). The red arrows point to the residues with non-β-sheet dihedral angles.

In recent years, labile amyloids formed by proteins with low complexity domains (LCDs), have attracted new attention as a structure contributing to the formation of membraneless organelles. LCD is a domain in proteins with little diversity in amino acid composition, and many intrinsically disordered regions [79]. There have been an increasing number of reports suggesting that RNA-binding proteins consisting of stress granules, such as fused-in sarcoma (FUS), heterogeneous nuclear ribonucleoprotein A1/A2 (hnRNPA1/A2), and TDP-43 tend to form labile and reversible amyloids with LCDs within their protein sequences, probably acting as an adhesive glue that mediates the assembly of membraneless organelles [79,80,81,82]. The LCDs of these RNA-binding proteins are rich in polar residues, aromatic residues, and glycine residues, and they contain a large number of (G/S-Y/F-G/S) motifs (Figure 8a). Due to their similarity to the amino acid sequences of the prion domain of yeast prion proteins, the LCDs of these RNA-binding proteins referred to as prion-like LCDs.

The above RNA-binding proteins have three types of self-association states assembled through their LCDs, i.e., the phase-separated droplets, hydrogel, and irreversible amyloids, and labile amyloids appear to be involved in the former two states. The labile amyloid structures are metastable, change occasionally to irreversible amyloids, and accumulate in the cytoplasm or nucleus as inclusions in neurodegenerative diseases including amyotrophic lateral sclerosis (ALS), frontotemporal dementia (FTD), or multisystem proteinopathy (MSP) [83,84,85]. Either the full-length proteins or isolated LCDs of these RNA-binding proteins cause liquid–liquid phase separation (LLPS), which involves the membraneless organelle assembly [79,80,81,82]. Since the phase-separated droplets are retained by the weak transient interactions between the LCDs, the proteins in droplets have liquid-like fluidity, despite their highly concentrated state. The structural details of LCDs within the droplet remain controversial. The LCDs of FUS within phase-separated droplets have been shown to retain their disordered structure using solution NMR [86]. On the other hand, studies using the chemical footprinting method suggest that the LCDs of TDP-43 have cross-β structures when forming droplets [87]. In the case of hnRNPA2, there are suggestions of both disordered structure and cross-β structure [88,89], which may suggest that LCD structures are sensitive to experimental conditions.

LLPS by proteins with LCDs sometimes leads to hydrogel formation. Although hydrogels are shown to be of diminished fluidity compared to the droplet state of their constituting proteins, they can return easily to solution-like, phase-separated droplet states in response to environmental stimuli [79,90,91,92]. The LCDs of constituent proteins have an amyloid-like cross-β structure composed of weak intermolecular interactions, i.e., labile amyloids, in the hydrogel. Labile amyloids dynamically assemble and disassemble in response to temperature and other stimuli [79,93]. In hnRNPA1, removing the labile amyloid core segment induced a decrease in stress granules recruitment, indicating that reversible labile amyloids are also involved in membraneless organelle assembly or stabilization, in addition to the phase separated droplets [94]. The reversible labile amyloid conformation of the LCDs of FUS and hnRNPA2 were revealed by solid-state NMR and cryo-electron microscopy, respectively [93,95]. The ordered amyloid core regions have 57 residues out of LCD 214 residues of FUS [95], and 57 residues out of LCD 161 residues for hnRNPA2 [93]. The β-sheet content of the amyloid cores are 26% (FUS) and 23% (hnRNPA2), which is much lower than those of pathogenic amyloids (>70%) [93]. However, the isolated C-terminal LCD (111–214), which does not form an amyloid core in the full-length FUS LCD, has been shown to have strong propensity to form a stable cross-β structure [96]. This suggests that the LCD sequence has a mechanism to acquire the labile and reversible nature by limiting the structuring of regions with a strong propensity for amyloid formation.

Recently, Eisenberg and co-workers succeeded in isolating and analyzing the crystal structures of the short segments forming reversible amyloids from the LCDs of FUS, hnRNPA1, nup98, and TDP-43 [97,98]. The structures revealed kinked β-sheets, in which kinks occur at the location of the glycine or aromatic residues within the segment. These short segments are named LARKS (low-complexity aromatic-rich kinked segments) for their structural characteristics. While aromatic side chains typically contribute to the intra-sheet and inter-sheet stabilization, those at the kinks prevent side chains from forming steric zipper structures at the β-sheet interface [97,98]. At the same time as the discovery of LARKS, Liu, Li and co-workers isolated short segments capable of forming reversible amyloids from FUS and hnRNPA1, which they named RAC (reversible amyloid core). They also determined their structures using microelectron diffraction and X-ray diffraction, and revealed that the structure of the RAC from hnRNPA1 is kinked, like that of LARKS [94,99] (Figure 8b).

Furthermore, the amyloid structure formed by the RAC of hnRNPA1 has provided an intriguing suggestion that the continuous stacking of aspartate residues contributes to decreasing the stability of a cross-β structure [94,99]. The contribution of the charged residues seems similar to those found in the functional amyloids formed by β-endorphin and Orb2, and is also supported by certain types of mutations involving ALS/FTD and MSP that promote the formation of irreversible amyloids. For the FUS mutants S96del and G156E involved in ALS/FTD, it has been shown that the temperature-dependent reversibility of FUS hydrogel was weakened [92]. The hnRNPA2 mutant D290V is involved in MSP, the mutation site of which is located within the amyloid core region of the LCD, increasing amyloid stability and reducing reversibility by eliminating charge repulsion [93,100]. A similar mutation, D262V/N, which has been identified in familial ALS, has been shown to reduce amyloid reversibility in both the full-length hnRNPA1 and hnRNPA1 short segments [94]. However, it should be noted that the introduction of charged groups does not always provide the dynamic feature of amyloid fibrils. As seen in an ALS-linked mutant A315E of TDP-43, the introduction of charged groups may increase the stability of the aggregates in the short segments, resulting in deteriorated reversibility [97].

## 6. Conclusions and Future Perspectives

We have overviewed recent advances in the understanding of the structural features of amyloid fibrils based on previous observations and arguments. Numerous studies conducted over the last few decades have revealed a great deal of knowledge on the structure, stability, and formation mechanisms of amyloid fibrils. Remarkable developments in solid-state NMR and cryo-EM have had a significant impact on structure determination at the amino-acid-residue level, and have increased the number of determined amyloid fibril structures within the last five years. The accumulated literature has revealed the attractive potential of amyloid structures for the rational design of self-assembled nanomaterials. Furthermore, it is becoming clearer that the varied arrangements and packing manners of side chains contribute significantly to varied nanomechanical properties, leading to the various thermodynamic and kinetic properties of amyloid fibrils. The development of the quantitative indicators for assessing side-chain packing will advance our strategic control of the physical properties of amyloid structures.

Amyloid structures were initially recognized as an aberrant structural state harmful to biological systems. However, the discovery of functional amyloids changed this view and made us aware of the possibility of using them while controlling the structure. Furthermore, the labile amyloids involved in LLPS formation have pushed amyloid research to a new level with a focus on the dynamic and reversible features of amyloid structures. They are also expected to pave the way for new designs of amyloid-based nanostructures to achieve spatiotemporal control of their formation and disappearance. Structural polymorphism is another attractive feature of amyloid structure, although it is observed predominantly in disease-related amyloids, and rational control of polymorphism will allow for the strategic development of various mechanical and thermodynamic properties from a single amino-acid sequence. These characteristic features of the amyloid structures exhibited by natural amyloid fibrils will provide many implications for the use of amyloid structure and lead to future breakthroughs in protein engineering.

## Data Availability

Not applicable.
